# Neurodevelopmental Disorders and Prenatal Residential Proximity to Agricultural Pesticides: The CHARGE Study

**DOI:** 10.1289/ehp.1307044

**Published:** 2014-06-23

**Authors:** Janie F. Shelton, Estella M. Geraghty, Daniel J. Tancredi, Lora D. Delwiche, Rebecca J. Schmidt, Beate Ritz, Robin L. Hansen, Irva Hertz-Picciotto

**Affiliations:** 1 Department of Public Health Sciences, University of California, Davis, Davis, California, USA; 2 Division of General Medicine,; 3 Department of Pediatrics, and; 4 Center for Healthcare Policy and Research, School of Medicine, University of California, Davis, Sacramento, California, USA; 5 Department of Epidemiology,; 6 Department of Environmental Health Sciences, and; 7 Department of Neurology, Fielding School of Public Health and School of Medicine, University of California, Los Angeles, Los Angeles, California, USA; 8 UC Davis MIND (Medical Investigations of Neurodevelopmental Disorders) Institute, Sacramento, California, USA

## Abstract

Background: Gestational exposure to several common agricultural pesticides can induce developmental neurotoxicity in humans, and has been associated with developmental delay and autism.

Objectives: We evaluated whether residential proximity to agricultural pesticides during pregnancy is associated with autism spectrum disorders (ASD) or developmental delay (DD) in the Childhood Autism Risks from Genetics and Environment (CHARGE) study.

Methods: The CHARGE study is a population-based case–control study of ASD, DD, and typical development. For 970 participants, commercial pesticide application data from the California Pesticide Use Report (1997–2008) were linked to the addresses during pregnancy. Pounds of active ingredient applied for organophophates, organochlorines, pyrethroids, and carbamates were aggregated within 1.25-km, 1.5-km, and 1.75-km buffer distances from the home. Multinomial logistic regression was used to estimate the odds ratio (OR) of exposure comparing confirmed cases of ASD (*n* = 486) or DD (*n* = 168) with typically developing referents (*n* = 316).

Results: Approximately one-third of CHARGE study mothers lived, during pregnancy, within 1.5 km (just under 1 mile) of an agricultural pesticide application. Proximity to organophosphates at some point during gestation was associated with a 60% increased risk for ASD, higher for third-trimester exposures (OR = 2.0; 95% CI: 1.1, 3.6), and second-trimester chlorpyrifos applications (OR = 3.3; 95% CI: 1.5, 7.4). Children of mothers residing near pyrethroid insecticide applications just before conception or during third trimester were at greater risk for both ASD and DD, with ORs ranging from 1.7 to 2.3. Risk for DD was increased in those near carbamate applications, but no specific vulnerable period was identified.

Conclusions: This study of ASD strengthens the evidence linking neurodevelopmental disorders with gestational pesticide exposures, particularly organophosphates, and provides novel results of ASD and DD associations with, respectively, pyrethroids and carbamates.

Citation: Shelton JF, Geraghty EM, Tancredi DJ, Delwiche LD, Schmidt RJ, Ritz B, Hansen RL, Hertz-Picciotto I. 2014. Neurodevelopmental disorders and prenatal residential proximity to agricultural pesticides: the CHARGE study. Environ Health Perspect 122:1103–1109; http://dx.doi.org/10.1289/ehp.1307044

## Introduction

California is the top agriculture-producing state in the nation, grossing $38 billion in revenue from farm crops in 2010 (California Department of Food and Agriculture 2010). Each year approximately 200 million pounds of active pesticide ingredients are applied throughout the state [California Department of Pesticide Regulation (CDPR) 2014]. Although pesticides are critical for the modern agricultural industry, certain commonly used pesticides have been associated with abnormal and impaired neurodevelopment in children (Bouchard et al. 2010, 2011; Engel et al. 2007; Eskenazi et al. 2006; Grandjean et al. 2006; Guillette et al. 1998; Rauh et al. 2006; Ribas-Fito et al. 2006; Torres-Sánchez et al. 2007; Young et al. 2005). In addition, specific associations have been reported between agricultural pesticides and autism spectrum disorders (ASD) (Roberts et al. 2007) and the broader diagnostic category under which autism falls, the pervasive developmental disorder (PDD) (Eskenazi et al. 2007).

Developmental delay (DD) refers to significant delays young children experience reaching milestones in relation to cognitive or adaptive development. Adaptive skills include communication, self-care, social relationships, and/or motor skills. In the United States, DD affects approximately 3.9% of all children 3–10 years of age, and is approximately 1.7 times more common among boys than girls (Boyle et al. 2011).

Autism is a developmental disorder with symptoms appearing by 3 years of age. Specific deficits occur in domains of social interaction and language, and individuals show restricted and repetitive behaviors, activities, or movements (American Psychiatric Association 2000). The ASDs represent lower severity, usually with regard to language ability. ASDs affect boys 4–5 times more than girls, and the Centers for Disease Control and Prevention (2012) recently estimated a prevalence of 1.1% among children 8 years of age, a 78% increase since their 2007 estimate. Available evidence suggests that causes of both ASD and DD are heterogeneous and that environmental factors can contribute strongly to risk (Hallmayer et al. 2011; Mendola et al. 2002).

The majority of pesticides sold in the United States are neurotoxic and operate through one of three primary mechanisms: *a*) inhibition of acetylcholinesterase (AChE), *b*) voltage-gated sodium channel disruption, and/or *c*) inhibition of gamma-aminobutyric acid (GABA) (Casida 2009). AChE primarily functions as an inhibitory neurotransmitter, but also has critical roles in the development of learning, cognition, and memory. GABA is also an inhibitory neurotransmitter, and is necessary for development and maintenance of neuronal transmission.

Although limited research has assessed *in utero* exposures to pesticides, animal models (rats) of early exposure to organophosphates showed more severe neurodevelopmental effects for males than for females (Levin et al. 2001, 2010). On the basis of previously published epidemiology or mechanistic considerations, we selected the following pesticide families to investigate for this analysis: organophosphates, carbamates, organochlorines, and pyrethroids. Potential mechanisms linking these select pesticide groups to autism pathophysiology were recently reviewed (Shelton et al. 2012).

The aim of this paper was to explore the relationship between agricultural pesticide applications and neurodevelopmental outcomes by *a*) assessing the gestational exposure during pregnancy to CHARGE (Childhood Autism Risks from Genes and Environment) study mothers, *b*) testing the hypothesis that children with ASD or DD had higher risk of exposure *in utero* than typically developing children, and *c*) evaluating specific windows of vulnerability during gestation. Because of the well-defined case and control populations in the CHARGE study and the comprehensive availability of potential confounders, this analysis serves as exploratory research to identify environmental risk factors for ASD and DD, and contributes to a broader understanding of the potential risks to neurodevelopment from agricultural pesticides in a diverse population of California residents.

## Methods

*Study design*. The CHARGE study is an ongoing California population-based case–control study that aims to uncover a broad array of factors contributing to autism and developmental delay (Hertz-Picciotto et al. 2006). Since 2003, the CHARGE study has enrolled > 1,600 participants whose parents answer extensive questionnaires regarding environmental exposures including their place of residence during pregnancy. Here we report on ASD and DD in relation to gestational residential proximity to agricultural pesticide applications. The group of children with ASD includes approximately two-thirds with a diagnosis of full-syndrome autism or autistic disorder (68%) and one-third with a diagnosis of an autism spectrum disorder (32%).

Cases were recruited from children diagnosed with full-syndrome ASD or DD in one of the regional centers of the California Department of Developmental Services (DDS). Eligibility in the DDS system does not depend on citizenship or financial status, and is widely used across socioeconomic levels and racial/ethnic groups. It is estimated that 75–80% of the total population of children with an autism diagnosis are enrolled in the system (Croen et al. 2002). In addition to recruitment through the regional centers, some CHARGE participants are also recruited through referrals from other clinics, self-referral, or general outreach. The referents are recruited from the general population (GP) identified through California birth records, and are frequency matched to the autism case population on sex, age, and the catchment area for the regional they would have gone to, had they been a case. Children are eligible if they are 2–5 years of age, born in California, live with a biological parent who speaks either English or Spanish, and reside in the study catchment area. Currently, the catchment area for the CHARGE study participants consists of a 2-hr drive from the Sacramento area, but previously included participants from Southern California. Early in the study, recruitment in Southern California was terminated due to logistical difficulties that led to lower enrollment of general population controls.

Parents of children coming into the study with a previous diagnosis of ASD are administered the Autism Diagnostic Interview–Revised (ADI-R; Le Couteur et al. 2003; Lord et al. 1994, 1997), surveyed regarding a wide range of environmental exposures, and asked to report all addresses where they lived from 3 months before conception to the time of the interview.

Participating children are administered the Autism Diagnostic Observation Schedule (ADOS; Lord et al. 2000, 2003), which, combined with the ADI-R, is used to either confirm their diagnosis or reclassify them for purposes of our study. To rule out ASD, children who enter the study without an ASD diagnosis (from the DD or GP groups) are given the Social Communications Questionnaire (SCQ) (Rutter et al. 2003). Children with a previous diagnosis of DD are evaluated on both the Mullen Scales of Early Learning (MSEL) (Mullen 1995) and Vineland Adaptive Behavioral Scale (VABS) (Sparrow 2005). DD is confirmed if they scored ≥ 15 on the SCQ and ≤ 2 SDs lower than the mean (< 70) on the composite scores of MSEL and VABS. Those meeting criteria for one test, scoring < 77 on the other, and not qualifying for ASD, are classified as atypical and combined with the DD group (25 of the 168) for this analysis. For this sample, of those who entered the study as typically developing, 26 were reclassified with DD and 2 with ASD. Of those who entered as DD, 36 were reclassified with ASD. Only cases with completed diagnostic testing were included in the analysis presented here. Additional details on CHARGE study protocols have been published elsewhere (Hertz-Picciotto et al. 2006).

This study was approved by the institutional review boards for the State of California and the University of California. Written informed consent was obtained by the parent or guardian before collection of any data.

*Estimation of pesticide exposures*. Since 1990, California has required commercial application of agricultural pesticides to be reported to the CDPR, which makes data publically available in the form of the annual Pesticide Use Report (PUR). As described by CDPR (2014), the pesticide use report data includes

pesticide applications to parks, golf courses, cemeteries, rangeland, pastures, and along roadside and railroad rights-of-way. In addition, all postharvest pesticide treatments of agricultural commodities must be reported along with all pesticide treatments in poultry and fish production as well as some livestock applications. The primary exceptions to the reporting requirements are home-and-garden use and most industrial and institutional uses.

The PUR database includes all commercial applications at the county level, requiring spatially explicit (latitude and longitude) reporting for commercial agricultural applications. The PUR database then compiles agricultural pesticide applications throughout the state by square-mile areas (1.0 m^2^ or 2.6 km^2^) set by the U.S. Geological Survey, referred to as a meridian-township-range-section (MTRS). The amount of chemical applied is assigned to an MTRS by date, in pounds (each pound is 0.45 kg) of active ingredient only, excluding synergists and other compounds in the formulation. Mapping software (ArcGIS v10.0; ESRI, Redlands, CA) was used to create a geographic centroid (center-most point in the square mile) for each MTRS for use in this analysis.

From the CHARGE questionnaire administered to the parent, residential addresses were collected and assigned for each day of the pre-conception and pregnancy periods, beginning 3 months before conception and ending with delivery, thereby accounting for participants who changed residences during that time. Addresses were manually cleaned for spelling errors and standardized in ZIP+4 software (http://www.semaphorecorp.com/). Of the 1,043 diagnostically evaluated participants at the time of this study, 983 had given address data for the time period of interest. Overall, 99% of addresses (970 participants with 1,319 unique addresses) were successfully geocoded to obtain a longitude and latitude with a match rate of at least 80% in ArcMap (ArcGIS v10.0; ESRI) using the U.S. Rooftop search algorithm. Unmatched addresses (*n* = 5) or ties (*n* = 10) were manually matched to the most likely address.

Next, a spatial model was developed in ArcMap, which created three buffers of varying sizes around each residence with radii of 1.25 km, 1.5 km, and 1.75 km. Where the buffer intersected a centroid (or multiple centroids), the MTRS corresponding with that centroid was assigned to that residence; subsequently, pesticides applied in that MTRS (or multiple MTRSs) were considered exposures for that mother with the timing based on linking the date of application to the dates of her pregnancy. Each pregnancy therefore was assigned an exposure profile corresponding to applications made to the MTRS nearest the mother’s home and days of her pregnancy on which those applications occurred (for a visual representation of the exposure model, see Supplemental Material, Figure S1).

We classified chemicals in the PUR according to chemical structure as members of the organophosphate, carbamate, pyrethroid, or organochlorine classes of pesticides. Subclasses of pyrethroids were categorized as type 1 and type 2 because they induce distinct behavioral effects in animal studies (Agency for Toxic Substances and Disease Registry 2003; Breckenridge et al. 2009). In addition, chlorpyrifos, an organophosphate widely used in agriculture, was explored independently because previous research had associated higher levels of prenatal exposure with diminished psychomotor and mental development in children at 3 years of age (Rauh et al. 2006).

*Statistical analysis*. Most homes (~ 70%) received estimated agricultural pesticide exposure values of 0 because there was no pesticide applied within the buffer zone. For ease of interpretation, we created, for each time period, binary (1 = exposed vs. 0 = not exposed) indicators as independent variables. Multinomial (polytomous) multivariate logistic regression modeling with survey weights was used to estimate the association of prenatal residential proximity to applied pesticides with a binary exposure variable (1 = exposed vs. 0 = not exposed) and a 3-level case status outcome [ASD/DD/TD (typically developing)], using TD children as the reference group. Because it was the only chemical evaluated independently as opposed to an aggregated class of chemicals with varying toxicity levels, chlorpyrifos (an organophosphate) was evaluated both as a dichotomous (any exposure within the buffer area vs. none) and as a continuous variable (untransformed, per 100 lb). Separate models were run for each time period, for each pesticide class of interest, and for alternative residential buffer radii.

Potential confounders were first identified as *a*) variables that may influence one’s exposure to pesticides, and *b*) variables that are known to influence the risk of ASD or DD, with no requirement for statistical significance of the univariate association with either the exposure or outcome, but rather an initial evaluation of the relationship between those variables. Formal confounder identification and inclusion was assessed using the combined directed acyclic graph (DAG) and change-in-estimate (in this case, a 10% change in the β of the primary exposure variable in the regression model) criteria (Weng et al. 2009). The DAG was used to establish which variables could potentially confound the associations between ASD or DD and exposure to agricultural pesticides, and the change in estimate criteria was then used to exclude inclusion of those variables that induced minimal (< 10%) change in the β estimate. All other variables which were identified as confounders and met the criteria of a ≥ 10% change in the β were included in the final models.

During model selection, we tested the joint versus independent effects of two classes of pesticides (e.g., pyrethroids and organophosphates) in models that contained each independent variable (dichotomous) for the two pesticides and an interaction variable of those two dichotomous variables. We also explored the possibility that another pesticide was responsible for the observed association due to correlation between pesticides (i.e., if one class is applied, another is more likely to be applied in that same buffer zone) by treating other classes of pesticides as potential confounders.

Final models were adjusted for paternal education (categorical), home ownership (binary), maternal place of birth (United States, Mexico, or outside the United States and Mexico), child race/ethnicity (white, Hispanic, other), maternal prenatal vitamin intake (binary; taken during the 3 months before pregnancy through the first month), and year of birth (continuous). Prenatal vitamin consumption in this time window was found in previous work to have an inverse association with ASD, meaning that early prenatal vitamin intake may confer a lower risk of ASD (Schmidt et al. 2011). Other potential confounders explored but found not to satisfy criteria for confounding (based on inclusion in the DAG or the change in estimate criterion) were distance from a major freeway, maternal major metabolic disorders (diabetes, hypertension, and obesity), gestational age (days), latitude of residence, type of insurance used to pay for the delivery (public vs. private), maternal age, paternal age, and season of conception. Maternal age, though a known risk for ASD, does not differ significantly between cases and controls in the CHARGE study because the participating mothers of TD children are older than those of the GP (Table 1).

**Table 1:** Characteristics [*n* (%) or mean ± SD] of the CHARGE study population (*n* = 970).

Characteristic	ASD	Delayed	Typical	*p*-Value	
				ASD vs. TD	DD vs. TD
Total	486	168	316
Male	414 (85.2)	115 (68.5)	262 (82.9)	0.39	0.0003
Child’s age at enrollment (months)	36.7 ± 9.7	38.3 ± 8.9	36.9 ± 8.9	0.73	0.11
Childs race/ethnicity				0.12	< 0.0001
White	246 (50.6)	66 (39.3)	165 (52.2)
Hispanic	130 (26.8)	60 (35.7)	73 (23.1)
Other	110 (22.6)	41 (24.4)	78 (24.7)
Mother’s age (years)	31.3 ± 5.5	30.8 ± 6.6	31.1 ± 5.7	0.69	0.57
Father’s age (years)	33.9 ± 6.4	33.1 ± 7.8	33.5 ± 7.0	0.49	0.52
Mother’s education				0.12	< 0.0001
High school or less	67 (13.8)	51 (30.4)	46 (14.6)
Some college	197 (40.5)	68 (40.5)	100 (31.7)
College or professional	222 (45.7)	49 (29.2)	170 (53.8)
Father’s education				0.58	< 0.0001
High school or less	106 (21.8)	74 (44.1)	81 (25.6)
Some college	153 (31.5)	47 (27.9)	91 (28.8)
College or professional	225 (46.3)	44 (26.2)	144 (45.6)
Regional center/region				< 0.0001	0.01
Alta	174 (35.8)	82 (48.8)	131 (41.5)
North Bay	64 (13.2)	19 (11.3)	53 (16.8)
East Bay	81 (16.7)	17 (10.1)	65 (20.6)
Valley Mountain	85 (17.5)	38 (22.6)	49 (15.5)
Southern California	82 (16.9)	12 (7.1)	18 (5.7)
Maternal birth place				0.07	0.0003
In the USA	367 (75.5)	127 (75.60	259 (82.0)
In Mexico	38 (7.8)	28 (16.7)	22 (7.0)
Outside USA or Mexico	81 (16.7)	13 (7.7)	35 (11.1)
Year of birth				0.0003	0.49
1999–2003	348(71.6)	94 (56.0)	187 (59.2)
2004–2008	138 (28.4)	74 (44.1)	129 (40.8)
Homeowner	320 (65.8)	100 (59.5)	242 (76.6)	0.001	< 0.0001
Private health insurance	402 (82.7)	118 (70.2)	270 (85.4)	0.31	< 0.0001
Periconceptional prenatal vitamin	252 (52.0)	79 (53.0)	189 (59.8)	0.003	0.01
Known chromosomal abnormality	11 (2.3)	50 (32.7)	0 (0.0)	—	—

All statistical analyses were conducted using SAS software (version 9.3; SAS Institute Inc., Cary, NC). Odds ratios (ORs) and 95% confidence intervals (CIs) were estimated using multinomial (polytomous) logistic regression models (PROC SURVEYLOGISTIC) with “survey” weights. Frequency-matching factors (regional center, age, and sex of child) were included to adjust for sampling strata using a STRATA statement.

The weights we used in exposure frequency and multinomial models adjusted for differential probabilities of enrollment in the study by case or control groups (ASD, DD, and GP controls) and by social and demographic factors (child race/ethnicity, maternal age, maternal education, insurance payment type at birth, regional center, parity, and maternal birth place) that influence voluntary participation in a case–control study. These weights represent the inverse of the probability of participation, within case and demographically defined groups. Thus, the weighted frequency distributions and regression models more accurately represent findings generalizable to the broader recruitment pools from which participants were drawn.

## Results

During pregnancy, residences of the CHARGE study participants were distributed broadly throughout California, with the greatest concentrations in Sacramento Valley, followed by the San Francisco Bay area and Los Angeles. One-third lived within 1.5 km of an agricultural pesticide application from one of the four pesticide classes evaluated. ASD and TD groups had similar sociodemographic profiles, with some variation by regional center, prenatal vitamin intake, and maternal place of birth, and more ASD cases were recruited earlier in the study than DD or TD children (Table 1). As described in “Methods,” early in the study, challenges were encountered recruiting non-ASD participants in Southern California, resulting in a greater proportion of ASD participants relative to TD participants from that regional center. The DD case group, which was not matched, differed from the reference group on many characteristics, including sex, race/ethnicity, maternal birth place, regional center, maternal education, and paternal education and appears to be of substantially lower socioeconomic status than either the ASD or TD groups (Table 1). Age of the child at enrollment was similar between the ASD and DD groups compared with the TD groups.

In the CHARGE study population, of the pesticides evaluated, organophosphates were the most commonly applied agricultural pesticide near the home during pregnancy. In the group exposed to organophosphates within 1.5 km of the home, 21 unique compounds were identified, the most abundant of which was chlorpyrifos (20.7%), followed by acephate (15.4%), and diazinon (14.5%) (see Supplemental Material, Table S1). The second most commonly applied class of pesticides was the pyrethroids, one-quarter of which was esfenvalerate (24%), followed by lamda-cyhalothrin (17.3%), permethrin (16.5%), cypermethrin (12.8%), and tau-fluvalinate (10.5%). Of the carbamates, approximately 80% were methomyl or carbaryl, and of the organochlorines, 60% of all applications were dienochlor. Among those exposed, only one-third were exposed to a single compound over the course of the pregnancy.

In the unweighted study population, little difference in exposure proportion was apparent, yet once the survey weights were applied, both case populations had higher exposure proportions than the TD controls, indicating that factors associated with exposure were also associated with study participation (Table 2). Because the study weights reflect the distributions of the three recruitment strata (ASD, DD, and TD controls) in the pool from which they were drawn, differences between case and control participation by regional center catchment area likely accounts for this effect. For example, DD cases proportionally under-enrolled in the CHARGE study from the Valley Mountain regional center compared with the recruitment pool. Because the Valley region had the highest proportion of exposed participants, weights that accounted for the discrepancy between the proportions of DD cases enrolled from the Valley region would more accurately represent the population distribution of cases and controls.

**Table 2:** Exposure to pesticide applications (any vs. none) within 1.5 km of the home during the 3 months before conception through delivery according to outcome (ASD *n* = 486, DD *n* = 168, TD *n* = 316).

Exposure	ASD			DD			TD		
	*n*	Unweighted %	Weighted %	*n*	Unweighted %	Weighted %	*n*	Unweighted %	Weighted %
No agriculturally applied pesticides	342	70.4	70.1	124	73.8	66.9	219	69.3	72.2
Any agriculturally applied pesticides	144	29.6	29.9	44	26.2	33.0	97	30.7	27.8
Organophosphates	125	25.7	26.6	32	19.1	25.2	84	26.6	24.9
Chlorpyrifos	61	12.6	14.4	20	11.9	18.4	45	14.2	12.4
Pyrethroids	106	21.8	22.5	36	21.4	28.3	67	21.2	20.1
Type 1 pyrethroids	49	10.1	10.4	17	10.1	16.3	29	9.2	7.9
Type 2 pyrethroids	100	20.6	20.9	34	20.2	26.9	63	19.9	19.1
Carbamates	54	11.1	11.0	13	7.7	11.1	30	9.5	7.3
Organochlorines	24	4.9	4.9	4	2.4	3.9	10	3.2	3.3
The development and use of CHARGE survey weights were designed to correct for the nonsociodemographically representative participation, i.e., the differences in participants vs. nonparticipants with regard to key sociodemographic factors such as maternal education, insurance payment type, birth regional center, birth place of mother, and child race. Survey weights are based on the probability of participation in the study.									

By pounds applied, the amount of pyrethroids and organophosphates (continuous, unweighted) applied within 1.5 km of the home were strongly correlated with each other (ρ = 0.74, *p* < 0.0001) and to a lesser extent organophosphates with carbamates (ρ = 0.45, *p* = 0.01) and carbamates with pyrethroids (ρ = 0.44, *p* < 0.0001). Because of the low prevalence of organochlorines and type 1 pyrethroids, these were excluded from the analyses, and carbamate exposure, though evaluated for pregnancy (any vs. none), was not evaluated by trimester due to small cell sizes of exposed participants. Overall, exposure to pesticides during gestation was slightly more common for male children than for female children (31% vs. 26%, *p* = 0.004).

For exposure (any vs. none) during pregnancy, children with ASD were 60% more likely to have organophosphates applied nearby the home [1.25 km distance; adjusted OR (aOR) = 1.60; 95% CI: 1.02–2.51] than mothers of TD children. Children with DD were nearly 150% more likely to have carbamate pesticides applied near the home during pregnancy (1.25 km distance; aOR = 2.48; 95% CI: 1.04–5.91). Both of these associations lessened as the buffer size grew larger (Tables 3 and 4), lending support to an exposure–response gradient.

**Table 3:** Adjusted ORs^*a*^ (95% CIs) for ASD and residential proximity to agricultural pesticide applications (any vs. none) within prespecified buffers, by time period.^*b*^

Pesticide, buffer radius (km)	Pregnancy	Preconception	1st trimester	2nd trimester	3rd trimester
Organophosphates
1.25	1.60 (1.02, 2.51)	1.37 (0.76, 2.50)	1.53 (0.87, 2.68)	1.57 (0.87, 2.83)	1.99 (1.11, 3.56)
1.5	1.54 (1.00, 2.38)	1.38 (0.82, 2.31)	1.45 (0.88, 2.41)	1.85 (1.08, 3.15)	2.07 (1.23, 3.50)
1.75	1.26 (0.83, 1.92)	1.30 (0.80, 2.13)	1.02 (0.63, 1.65)	1.54 (0.93, 2.55)	1.99 (1.20, 3.30)
Chlorpyrifos
1.25	1.57 (0.82, 3.00)	1.07 (0.40, 2.89)	1.26 (0.52, 3.06)	2.55 (0.95, 6.84)	1.83 (0.72, 4.65)
1.5	1.66 (0.94, 2.93)	1.07 (0.46, 2.48)	1.32 (0.65, 2.70)	3.31 (1.48, 7.42)	1.78 (0.82, 3.87)
1.75	1.78 (1.05, 3.02)	1.25 (0.59, 2.65)	1.12 (0.58, 2.16)	2.63 (1.28, 5.41)	2.15 (1.04, 4.41)
Pyrethroids
1.25	1.34 (0.82, 2.20)	1.82 (0.92, 3.60)	1.59 (0.86, 2.96)	1.56 (0.83, 2.94)	1.64 (0.84, 3.19)
1.5	1.41 (0.89, 2.25)	1.82 (1.00, 3.31)	1.53 (0.88, 2.67)	1.69 (0.93, 3.06)	1.87 (1.02, 3.43)
1.75	1.27 (0.83, 1.96)	1.69 (0.97, 2.95)	1.14 (0.67, 1.91)	1.49 (0.87, 2.58)	1.83 (1.04, 3.23)
Type 2
1.25	1.40 (0.83, 2.34)	2.01 (0.97,4.16)	1.64 (0.85, 3.17)	1.29 (0.65,2.56)	1.51 (0.75, 3.05)
1.5	1.53 (0.94, 2.51)	1.98 (1.06, 3.71)	1.85 (1.01, 3.38)	1.45 (0.78, 2.73)	1.67 (0.87, 3.21)
1.75	1.30 (0.82, 2.05)	1.64 (0.92, 2.94)	1.32 (0.76, 2.29)	1.33 (0.75, 2.38)	1.56 (0.86, 2.84)
Carbamates^*c*^
1.25	1.37 (0.66, 2.84)	—	—	—	—
1.5	1.80 (0.81, 3.08)	—	—	—	—
1.75	1.43 (0.78, 2.62)	—	—	—	—
^***a***^Multivariate multinomial conditional logistic regression with survey weights and strata variables for matching variables. All models were adjusted for paternal education, home ownership, maternal place of birth, child race/ethnicity, maternal prenatal vitamin intake (during the 3 months before pregnancy through the first month), and year of birth. ^***b***^Pregnancy: conception (day 0) to the end of pregnancy; preconception: 90 days before conception; 1st trimester: 0–90 days; 2nd trimester: 91–180 days; 3rd trimester: 181 days–birth. ^***c***^Due to low frequency of exposure, the cell counts were too small (< 10) to explore temporal associations, and thus are not presented here.					

**Table 4:** Adjusted ORs^*a*^ (95% CIs) for DD and residential proximity to agricultural pesticide applications (any vs. none) within prespecified buffers, by time period.^*b*^

Pesticide, buffer radius (km)	Pregnancy	Preconception	1st trimester	2nd trimester	3rd trimester
Organophosphates
1.25	1.23 (0.65, 2.31)	1.20 (0.54, 2.65)	1.29 (0.60, 2.79)	1.62 (0.75, 3.48)	1.10 (0.46, 2.67)
1.5	1.07 (0.60, 1.92)	0.94 (0.45, 1.97)	1.00 (0.50, 1.99)	1.46 (0.72, 2.96)	0.92 (0.40, 2.13)
1.75	1.01 (0.59, 1.73)	1.30 (0.69, 2.46)	0.98 (0.54, 1.80)	1.52 (0.81, 2.85)	1.21 (0.60, 2.46)
Chlorpyrifos
1.25	1.62 (0.68, 3.85)	1.73 (0.58, 5.17)	1.61 (0.53, 4.87)	1.73 (0.48, 6.19)	1.04 (0.25, 4.28)
1.5	1.31 (0.61, 2.82)	1.11 (0.41, 3.00)	1.27 (0.48, 3.36)	1.43 (0.46, 4.44)	0.73 (0.21, 2.48)
1.75	1.63 (0.84, 3.16)	1.34 (0.55, 3.25)	1.40 (0.62, 3.17)	1.63 (0.61, 4.39)	1.34 (0.50, 3.60)
Pyrethroids
1.25	1.53 (0.81, 2.90)	1.96 (0.90, 4.29)	1.70 (0.80, 3.61)	1.63 (0.72, 3.68)	1.69 (0.74, 3.88)
1.5	1.37 (0.76, 2.47)	1.44 (0.69, 3.03)	1.41 (0.72, 2.76)	1.27 (0.58, 2.79)	1.75 (0.81, 3.78)
1.75	1.19 (0.68, 2.08)	1.88 (0.98, 3.60)	1.36 (0.73, 2.51)	1.42 (0.72, 2.80)	2.34 (1.18, 4.67)
Type 2
1.25	1.56 (0.81, 2.90)	1.43 (0.61, 3.33)	1.60 (0.72, 3.59)	1.78 (0.78, 4.08)	1.80 (0.77, 4.18)
1.5	1.46 (0.79, 2.70)	1.09 (0.48, 2.46)	1.49 (0.71, 3.12)	1.41 (0.64, 3.13)	1.87 (0.85, 4.11)
1.75	1.34 (0.76, 2.37)	1.18 (0.57, 2.43)	1.37 (0.71, 2.64)	1.66 (0.84, 3.28)	2.31 (1.15, 4.66)
Carbamates^*c*^
1.25	2.48 (1.04, 5.91)	—	—	—	—
1.5	1.65 (0.70, 3.89)	—	—	—	—
1.75	1.32 (0.60, 2.88)	—	—	—	—
^***a***^Multivariate multinomial conditional logistic regression with survey weights and strata variables for matching variables. All models were adjusted for paternal education, home ownership, maternal place of birth, child race/ethnicity, maternal prenatal vitamin intake (during the 3 months before pregnancy through the first month), and year of birth. ^***b***^Pregnancy: conception (day 0) to the end of pregnancy; preconception: 90 days before conception; 1st trimester: 0–90 days; 2nd trimester: 91–180 days; 3rd trimester: 181 days–birth. ^***c***^Due to low frequency of exposure, the cell counts were too small (< 10) to explore temporal associations, and thus are not presented here.					

Examining specific gestational time windows, associations with pesticide applications of organophosphates and pyrethroids suggested an association between second- and third-trimester exposure to organophosphates and ASD, and preconception and third-trimester pyrethroid exposure (Table 3). Although those time periods describe the statistically significant associations, many of the effect estimates tended away from the null, which indicates a lack of precision in the specificity of any one time period and compound presented here.

For DD, the sample size permitted only temporal associations to be evaluated for organophosphates and pyrethroids, which were mostly > 1 (the null value), but only one statistically significant association was detected for third-trimester pyrethroid applications. In general, likely because a smaller sample of DD cases was exposed to agricultural pesticides, the estimates had a lower level of precision than the ASD case group. In addition, although carbamates were associated with DD for applications during pregnancy, the sample of exposed cases was too small to evaluate by trimester (Table 4).

For models evaluating the exposure to chlorpyrifos as a continuous variable with all other covariates remaining the same as above models, each 100-lb (45.4 kg) increase in the amount applied over the course of pregnancy (within 1.5 km of the home) was associated with a 14% higher prevalence of ASD (aOR = 1.14; 95% CI: 1.0, 1.32), but no association was detected with DD. Because aggregate classes of chemical do not have a uniform toxicity, we did not examine the pounds of classes (e.g., organophosphates) of chemicals as a continuous variable because compounds with a higher toxicity may be applied in lower volumes.

The role of simultaneous exposure to multiple classes of pesticides was evaluated in post hoc analyses. First, we evaluated combined categories of organophosphates and pyrethroids, organophosphates and carbamates, and pyrethroids or carbamates as a 3-level variable (0 = unexposed, 1 = exposed to one or the other, and 2 = exposed to both). However, effects from multiple exposures were not found to be higher than the observations of the individual classes of pesticides. Second, we adjusted models of one pesticide for the other. In models for organophosphates, adjusting for pyrethroids attenuated the third-trimester association with ASD slightly, but not substantially (< 10% change in β estimate) (data not shown). In additional analyses, we evaluated the sensitivity of the estimates to the choice of buffer size, using four additional sizes between 1 and 2 km: Results and interpretation remained stable (data not shown).

## Discussion

Applications of two of the most common agricultural pesticides (organophosphates and pyrethroids) nearby the home may increase the prevalence of ASD. Specifically, we observed positive associations between ASD and prenatal residential proximity to organophosphate pesticides in the second (for chlorpyrifos) and third trimesters (organophosphates overall), and pyrethroids in the 3 months before conception and in the third trimester. Our findings relating agricultural pesticides to DD were less robust, but suggested an associated with applications of carbamates during pregnancy nearby the home. Because pesticide exposure is correlated in space and time, differences in time windows of vulnerability, if they exist, may be difficult to detect, and variation in associations according to time window of exposure may not represent causal variation.

These findings support the results of two previous studies linking ASD to gestational agricultural pesticide exposure. Using data from the California Department of Developmental Services and California Birth Records, Roberts et al. (2007) conducted a case–control study of 465 cases of autism and 6,975 controls. Although their main finding was an association between ASD and residential proximity to organochlorine compound applications (which we could not evaluate due to low exposure prevalence of this chemical class), they also reported associations with gestational exposures to organophosphates [β = 0.462, *p*-value 0.042 (confidence interval not reported) and bifenthrin (β = 1.57, *p*-value = 0.049 (confidence interval not reported)], a pyrethroid pesticide (Roberts et al. 2007). Eskenazi et al. (2007) found a relationship between symptoms of PDD and prenatal urinary metabolites of organophosphates in a cohort study (CHAMACOS; Center for the Health Assessment of Mothers and Children of Salinas) of mothers living in the Salinas valley. Each 10-fold increase in these metabolites doubled the odds (OR = 2.3, *p* = 0.05) of PDD at 2 years of age; postnatal concentrations showed some association as well (OR = 1.7, *p* = 0.04) (Eskenazi et al. 2007). Several studies have also reported evidence of an interaction between organophosphate exposure and polymorphisms for the *PON1* gene, which codes for the enzyme paroxonase 1, in relation to neurodevelopment (Costa et al. 2005; D’Amelio et al. 2005; Furlong et al. 2005; Lee et al. 2013).

With regard to DD, several studies have reported associations of pesticide exposures with continuous scores on specific cognitive tests. For example, in a cross-sectional study of 72 children < 9 years of age in Ecuador, those prenatally exposed to pesticides as assessed by maternal occupation in the floriculture industry during pregnancy performed worse on the Stanford-Binet copying test than did children whose mothers did not work in floriculture during pregnancy (Grandjean et al. 2006). In another study of maternal occupation in the flower industry, exposed children performed worse on tests of communication, visual acuity, and fine motor skills, with delays of 1.5–2 years in reaching normal developmental milestones (Handal et al. 2008). In the CHAMACOS cohort, organophosphate urinary metabolites from the first and second halves of pregnancy were associated with an average deficit of 7.0 IQ points, comparing the highest quintile to the lowest (Bouchard et al. 2011). A study of inner city children at 3 years of age found that those with the highest (vs. lowest) umbilical cord concentrations of chlorpyrifos were 5 times more likely to have delayed psychomotor development and 2.4 times more likely to have delayed mental development as assessed by cut-off values of continuous scores on the Bayley Scales of Infant Development-II (Rauh et al. 2006).

Strengths of this study include well-defined case and control populations confirmed by standardized diagnostic instruments, extensive information on covariates, and a thorough confounder identification and control strategy. Because children can overcome developmental delay, or may move in or out of the ASD case definition over time, diagnostic confirmation at enrollment minimized outcome misclassification. Further, collection of information on all addresses during pregnancy likely reduced exposure misclassification, because 20% of the population had moved at least once during pregnancy.

Several limitations to this study were unavoidable in the exposure assessment, potentially producing misclassification. Primarily, our exposure estimation approach does not encompass all potential sources of exposure to each of these compounds: Among them were external nonagricultural sources (e.g., institutional use, such as around schools); residential indoor use; professional pesticide application in or around the home for gardening, landscaping or other pest control; and dietary sources (Morgan 2012). Other sources of potential error include errors in reporting to the PUR database, the assumption of homogeneity of exposure within each buffer, and potential geocoding errors. Seasonal variation and address changes mid-pregnancy were accounted for by assigning an address to each day instead of one address for the individual, but information on hours spent in the home or elsewhere was not available.

Use of the PUR data has been refined by some researchers who have enhanced the 1-mi^2^ resolution of the PUR data by incorporating land use data (Nuckols et al. 2007; Rull and Ritz 2003). This approach demonstrates higher correlation of PUR-based exposure estimates with in-home carpet dust pesticide concentrations than the PUR data alone (Gunier et al. 2011). In our case, land use reports were not available for about half the CHARGE study counties; given an already low prevalence of exposure, the loss of power by excluding those counties would have outweighed any benefit of increased specificity in exposure estimates from land use data.

Although organophosphate use drastically increased between the 1960s through the late 1990s (U.S. Department of Agriculture 2006), over the past decade, use has been declining (U.S. Environmental Protection Agency 2011). For indoor use, chlorpyrifos has largely been replaced with pyrethroids (Williams et al. 2008), but research indicates pyrethroids may not necessarily be safer. In an *in vitro* study comparing the toxicity of a common pyrethroid, cyfluthrin, with chlorpyrifos, at the same doses cyfluthrin induced either an equivalent or higher toxic effect on the growth, survival, and function of primary fetal human astrocytes, and induced inflammatory action of astrocytes that can mediate neurotoxicity (Mense et al. 2006). In another *in vitro* study comparing the neurotoxicity of fipronil to chlorpyrifos, fipronil induced more oxidative stress and resulted in lower cell counts for nondifferentiated PC12 cells than chlorpyrifos, and disrupted cell development at lower thresholds, leading the authors to conclude that fipronil was in fact more detrimental to neuronal cell development than chlorpyrifos (Lassiter et al. 2009). Although further studies are underway, because of the observed associations in humans and direct effects on neurodevelopmental toxicity in animal studies, caution is warranted for women to avoid direct contact with pesticides during pregnancy.

## Conclusions

Children of mothers who live near agricultural areas, or who are otherwise exposed to organophosphate, pyrethroid, or carbamate pesticides during gestation may be at increased risk for neurodevelopmental disorders. Further research on gene–environment interactions may reveal vulnerable subpopulations.
