# Erratum: “Neurodevelopmental Disorders and Prenatal Residential Proximity to Agricultural Pesticides: The CHARGE Study”

**DOI:** 10.1289/ehp.122-A266

**Published:** 2014-10-01

In “Neurodevelopmental Disorders and Prenatal Residential Proximity to Agricultural Pesticides: The CHARGE Study” by Shelton et al. [Environ Health Perspect 122:1103–1109 (2014); doi:10.1289/ehp.1307044], the following disclaimer was inadvertently omitted from the Competing Financial Interest Declaration:

D.J.T., R.J.S., R.L.H., and I.H.-P. have received travel reimbursements and grant support from Autism Speaks, an autism advocacy group. Further, the authors state that their freedom to design, conduct, interpret, and publish research is not compromised by any controlling sponsor as a condition of review and publication.

The authors regret the error.

